# Optimization of a synthetic mixture composed of major *Trichoderma reesei *enzymes for the hydrolysis of steam-exploded wheat straw

**DOI:** 10.1186/1754-6834-5-9

**Published:** 2012-02-28

**Authors:** Hélène Billard, Abdelaziz Faraj, Nicolas Lopes Ferreira, Sandra Menir, Senta Heiss-Blanquet

**Affiliations:** 1 IFP Energies nouvelles, Biotechnology Department, 1 et 4 Avenue de Bois-Préau, 92852 Rueil-Malmaison Cedex, France; 2 IFP Energies nouvelles, Applied Mathematics Department, 1 et 4 Avenue de Bois-Préau, 92852 Rueil-Malmaison Cedex, France

**Keywords:** *Trichoderma reesei*, cellulases, xylanase, wheat straw, enzymatic hydrolysis, experimental design

## Abstract

**Background:**

An efficient hydrolysis of lignocellulosic substrates to soluble sugars for biofuel production necessitates the interplay and synergistic interaction of multiple enzymes. An optimized enzyme mixture is crucial for reduced cost of the enzymatic hydrolysis step in a bioethanol production process and its composition will depend on the substrate and type of pretreatment used. In the present study, an experimental design was used to determine the optimal composition of a *Trichoderma reesei *enzyme mixture, comprising the main cellulase and hemicellulase activities, for the hydrolysis of steam-exploded wheat straw.

**Methods:**

Six enzymes, CBH1 (Cel7a), CBH2 (Cel6a), EG1 (Cel7b), EG2 (Cel5a), as well as the xyloglucanase Cel74a and the xylanase XYN1 (Xyl11a) were purified from a *T. reesei *culture under lactose/xylose-induced conditions. Sugar release was followed in milliliter-scale hydrolysis assays for 48 hours and the influence of the mixture on initial conversion rates and final yields is assessed.

**Results:**

The developed model could show that both responses were strongly correlated. Model predictions suggest that optimal hydrolysis yields can be obtained over a wide range of CBH1 to CBH2 ratios, but necessitates a high proportion of EG1 (13% to 25%) which cannot be replaced by EG2. Whereas 5% to 10% of the latter enzyme and a xylanase content above 6% are required for highest yields, these enzymes are predicted to be less important in the initial stage of hydrolysis.

**Conclusions:**

The developed model could reliably predict hydrolysis yields of enzyme mixtures in the studied domain and highlighted the importance of the respective enzyme components in both the initial and the final hydrolysis phase of steam-exploded wheat straw.

## Background

The production of bioethanol from plant biomass is seen as a possible strategy to reduce greenhouse gas emissions and the current dependence of industrialized nations on declining fossil fuels. Renewable lignocellulosic biomass is generally cheap and abundant and does not compete with food production as is the case for agricultural crops. Raw materials include wood residues, dedicated crops such as poplar or Miscanthus, agricultural residues and waste paper. Wheat straw is one of the most abundant crop residues in middle European countries with a production of over 130 million tons [1] and represents a low-cost source of lignocellulosic biomass.

In nature, plant cell wall carbohydrates are hydrolyzed to soluble sugars by (hemi)cellulolytic enzymes from both bacteria and fungi, the latter being very efficient degraders [2]. *Trichoderma reesei *is the major fungus used for industrial cellulase production. The secreted cellulases comprise two cellobiohydrolases and eight endoglucanases from six glycoside hydrolase families [3] which act in a synergistic manner to degrade the plant biomass, together with beta-glucosidases and hemicellulases. In total, the *T. reesei *genome contains 200 glycoside hydrolases and more than 20 of them can be found in the secretome [3,4]. It is supposed that the presence of multiple enzymes displaying the same type of activity (as is the case for endoglucanases) is necessary for efficient biomass hydrolysis, but the precise role of the individual enzymes is still not well understood.

An important number of research studies have been dedicated to the optimization of conversion processes from lignocellulose to fuel ethanol in the last decades resulting in significant progress [5-10]. Industrial bioethanol production processes usually include a physicochemical pretreatment of the lignocellulosic substrate, which aims at increasing the accessibility of the material to hydrolytic enzymes. One of the most efficient pretreatments for wheat straw is steam explosion which consists of heating the biomass with pressurized steam for a few minutes and then rapidly releasing pressure [11]. It hydrolyzes most of the hemicelluloses and part of the lignin present, liberating the access to cellulose fibers and rendering them more amenable to digestion. However, even if this substrate is relatively well hydrolyzed by a (hemi)cellulolytic enzyme cocktail at moderate enzyme loadings (about 90% hydrolysis in 72 hours), saccharification of lignocellulosic materials is generally still too inefficient to support a cost-efficient process [12,13]. A major problem in developing industrial enzyme mixtures is the different structure and composition of the potential substrates and naturally produced enzyme cocktails are often not adapted to efficiently degrade different pretreated materials. One strategy to improve the hydrolytic activity of the enzyme cocktail is, therefore, to adapt its composition to the substrate to be hydrolyzed.

Previous studies have shown that the efficiency of commercially available enzyme cocktails could be improved by adding xylanase [14-16]. An experimental design to optimize the hydrolysis of barley straw with the four major cellulases of *T. reesei *indicated that optimal ratios differed from the composition of a naturally produced cocktail, and showed that an optimized mixture of the three major enzymes, CBH1, CBH2 and EG1, could reach 80% of the hydrolysis yield obtained with a commercial enzyme preparation [17]. In order to achieve higher hydrolysis yields, the authors postulated the requirement of hemicellulases or accessory enzymes. Synthetic mixtures comprising more components (11 to 16), assayed on different substrates, such as corn stover, Miscanthus, switchgrass and poplar, could indeed equal the performance of complete *T. reesei *enzyme cocktails [18,19]. These studies also showed that the optimal composition varied greatly with the type of feedstock, pretreatment and substrate to enzyme ratio and revealed the difficulty of predicting the necessary enzyme components due to a lack of fundamental mechanistic understanding [19,20].

In the present study, we analyzed the hydrolysis of a single substrate, steam-pretreated wheat straw, by a six component mixture at different stages. An experimental mixture plan was set up for the four major *T. reesei *cellulases CBH1 (Cel7a), CBH2 (Cel6a), EG1 (Cel7b) and EG2 (Cel5a), as well as the xyloglucanase Cel74a and the xylanase XYN1 (Xyl11a) which were all purified from a complete *T. reesei *enzyme cocktail. A statistical model was established allowing the prediction of optimized mixtures for both initial conversion rates and final yields. In addition, the impact of a changing cocktail composition on both responses was assessed to understand better the role of the individual enzymes and their synergistic interactions. Results suggest that the studied enzymes have distinct and only partially redundant roles in initial and late hydrolysis stages of steam-pretreated wheat straw.

## Results and discussion

Purification of the *T. reesei *enzyme mixture produced by strain CL847 on an anionic exchange column allowed the recovery of six major fractions, representing the six major cellulolytic proteins found after induction by a lactose/xylose 70:30 mixture. Two-dimensional (2D) electrophoresis and activity measurements confirmed the purity and allowed unambiguous determination of the identity of each fraction. The specific activities of the six major enzymes obtained, the two cellobiohydrolases CBH1 (Cel7a) and CBH2 (Cel6a), the major two endoglucanases EG1 (Cel7b) and EG2 (Cel5a), as well as the xyloglucanase Cel74a and the xylanase XYN1, were measured on Avicel, carboxymethylcellulose (CMC), xylan and xyloglucan (Table 1). The two cellobiohydrolases have high activity on Avicel cellulose, consistent with values found in the literature [20,21] whereas endoglucanases and XYN1 show lower activities. A low activity of xylanase on Avicel and CMC has been reported previously [22]. As expected, highest activity on amorphous cellulose was seen for endoglucanases. CBH2 also has some activity on CMC, which has also been observed with the heterologously expressed *T. reesei *CBH2 enzyme and is consistent with the endo-type side-activity of this enzyme [23]. Besides XYN1 and Cel74a, EG1 also has xylanase activity consistent with previous results [24].

**Table 1:** Specific activities of purified enzymes on solid model substrates

	CBH1	CBH2	EG1	EG2	Cel74a	XYN1
Avicel	0.035	0.027	0.020	0.007	0.008	0.011
CMC	< 0.1	0.3	0.6	0.4	0.1	0.1
Xylan	ND	ND	1.6	ND	2.6	2.8
Xyloglucan	< 1	< 1	731	53	54	70

Released glucose equivalents were measured by the DNS method. Cellulase activities are expressed in units (μmol glucose equivalents released per minute and per mg enzyme). For xylanase and xyloglucanase activities, values correspond to μmol xylose equivalents min^-1 ^mg^-1^. CMC, carboxymethylcellulose; DNS, dinitrosalicylic acid; ND = not detectable.

**Figure 1:**
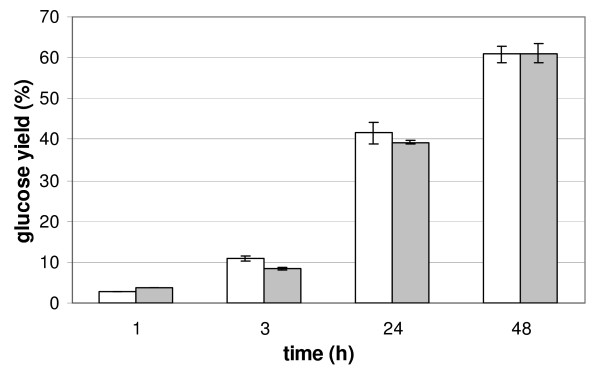
**Hydrolysis yields of steam-exploded wheat straw with *T. reesei *enzymes**. Hydrolysis was performed either with the complete *T. reesei *enzyme cocktail K630 (white bars), or the reconstituted cocktail (grey bars) composed of the six purified enzymes (CBH1 44%, CBH2 30%, EG17.3%, EG2 14.2%, Cel74a 0.5%, XYN1 4%). Substrate loading was 1% DM and enzymes were added at 2.5 mg/g substrate. DM, dry matter.

An artificial enzymatic cocktail comprising the six purified enzymes in the same proportion as the *T. reesei *K630 cocktail which was obtained after induction by a lactose:xylose (60:40) mixture was reconstituted. The hydrolysis yields on steam-exploded wheat straw of the reconstituted mixture was similar to that of the K630 cocktail, which indicates that the purified enzymes were highly active and which validates them for use in the following mixture experiments (Figure 1). The rather modest hydrolysis yield can be explained by the low enzyme loading (2.5 mg g^-1 ^dry matter); about 61% of available glucose was hydrolyzed in 48 hours. For comparison, 66% hydrolysis could be obtained using the commercial enzyme cocktail GC220 at the same enzyme loading and substrate consistency. With a higher enzyme loading (10 mg g^-1 ^dry matter) 100% of the cellulose were digested by GC220 in 48 hours and, at 15% consistency, 74% was hydrolyzed in 144 hours.

### Set-up of experimental design

In order to optimize the hydrolysis efficiency of a *T. reesei *cellulase mix on steam-pretreated wheat straw, the relative abundances of each of the six major enzymes were varied using an experimental design. The borders of the experimental domain were carefully chosen. Special attention was paid to avoid a too large domain as this may impact the reliability of predictions within the domain. On the other hand, it should not be too small and contain the optimum, since extrapolation outside the domain borders is impossible.

The lower and upper limits of each component were, therefore, determined following rational considerations:

1. Cellobiohydrolases are known to be important for cellulose hydrolysis [25] and the sum of CBH1 and CBH2 should constitute the majority of the enzyme cocktail (>50%). In addition, literature data showed that higher CBH2/CBH1 ratios are more beneficial for hydrolysis of steam-exploded wheat straw than lower ones [17]. Their relative abundances were thus varied from 10:1 to 1:3.

2. The presence of endoglucanases is necessary and EG1 and EG2 should each at least make up 2% of the mixture. A higher upper limit was chosen for EG1, as this enzyme was shown in preliminary experiments to be more important than EG2 for steam-exploded wheat straw (N. Lopes Ferreira, unpublished results).

3. Cel74a is a minor enzyme constituent and its upper limit was, therefore, fixed at 5%.

4. Steam-exploded wheat straw only contains a small amount of xylane (< 3%). However, recent studies have shown the importance of xylanases for lignocellulose hydrolysis [26,27]. A minimum of 3% xylanase was, therefore, imposed.

Table 2 shows the respective borders for all variables, upper limits for CBH1 and CBH2 being implicit by the other constraints.

**Table 2:** The constraints of the system

Enzyme	Variable in model	Lower limit (%)	Upper limit (%)
CBH1	*x*_1_	9	70
CBH2	*x*_2_	23	84
EG1	*x*_3_	2	30
EG2	*x*_4_	2	15
Cel74a	*x*_5_	0	5
XYN1	*x*_6_	3	6

### Modeling of results

The model presented by equation 1 (see Methods) delivers a response surface for the experimental design space, consisting of the predicted values of the response *y *in each point. That means that, if this model is validated, the response *y *in every point of the design space can be predicted [28]. In our case, five response *y*'s are calculated which correspond to the coefficients of equations 2 and 3, describing the hydrolysis reaction:

(2) $${\text{R}}_{1}\left(\text{t}\right)=v_{01}\text{t}+\frac{1}{2}\gamma_{1}{\text{t}}^{2}$$

(3) $${\text{R}}_{2}\left(\text{t}\right)={\text{R}}_{0}+v_{02}\text{t}+\frac{1}{2}\gamma_{2}{\text{t}}^{2}$$

The two polynomials represent two different time intervals: equation 2 describes the reaction from 0 to 6 hours and allows the calculation of the initial conversion rate v_01_, whereas equation 3 corresponds to the time course of conversion between 6 and 48 hours and allows the calculation of the final yield Rf (which is considered here to be the yield after 48 hours). Together with the acceleration (the rate of speed change as a function of time (%/h^2^), γ_1 _and γ_2_), and the initial conversion rate for phase 2, ν_02, _there are five response *y*'s (considered as experimental outputs of the process) to be calculated by the model.

**Figure 2:**
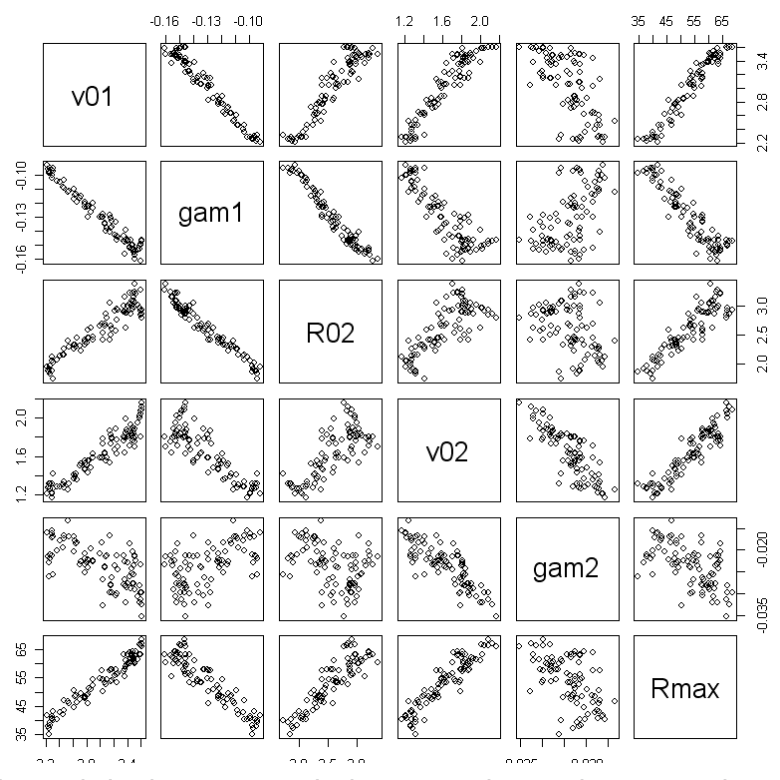
**Correlation of the six calculated responses: initial velocities ν_01 _and ν_02_, accelerations γ_1 _and γ_2_, intermediary yield R02 and final yield Rf**.

A correlation analysis with the five experimental response *y*'s shows that they are strongly correlated (Figure 2). Therefore, instead of modelling each response, only models for the responses ν_01 _and Rf were established. Applied to the hydrolysis reaction, this correlation means that enzyme mixtures showing a maximal initial conversion rate will globally also have a good final conversion yield. The link between the initial conversion rate ν_01 _and the final conversion yield Rf can be modelled by the two following equations:

(4) $$\text{Rf}=16.6^{*}\nu_{01}-5.09$$

(5) $$\nu_{01}=0.057^{*}\text{Rf}+0.48$$

Computation of coefficients of equation 1 led to the establishment of a predictive model. The results for ν_01 _and Rf are given in additional file 1. Some coefficients of the two models are not significant (*P*-values >0.005). The use of model reduction (by a stepwise technique) does not improve the R^2 ^of prediction of the two models, respectively equal to 0.89 and 0.92. We therefore decided to keep these models which are used in the following section to understand and optimize the hydrolysis process.

### Model validation

**Figure 3:**
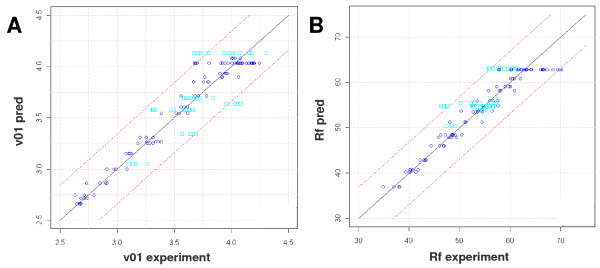
**Predicted versus experimental initial conversion rate (A) and final yield (B)**. Dark blue circles correspond to points used for model set up, light blue rectangles to validation points. Dashed lines delimit the area corresponding to 2 σ.

Crossplots of predicted and experimentally determined values for the initial conversion rate ν_01 _and the final yield Rf show that all points lie within the border of 2σ. This applies both to points used for establishment of the model and for validation points, underlining the good predictive capacity of the model (Figure 3). In order to determine the global experimental error, the point in the center was repeated nine times (each time in triplicate) and two other points were repeated three times. The resulting mean standard deviation was 3.2% for the hydrolysis yield and 0.2% hour^-1 ^for the initial conversion rate. Instead of reporting SD for each point which would be based on a single repeat in triplicate only, these global SD values are applied to all points.

**Figure 4:**
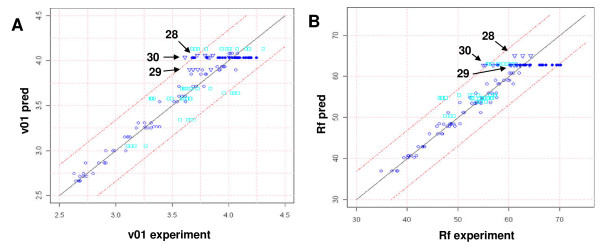
**Predicted versus experimental initial conversion rate (A) and final yield (B)**. Triangles show points with maximized v01 or Rf (points 28 to 30). Circles represent points used for model establishment, filled dark blue circles correspond to the central point (control). Open light blue rectangles are validation points. Rf, final yield.

Once statistically validated, the model was used to predict enzyme mixtures with the highest final yield and the highest initial conversion rate, respectively. Table 3 shows the composition of these points with values for ν_01 _and Rf. Predicted and experimentally determined values are again in good accordance and within the interval of experimental error (Figure 4). Experimentally determined initial conversion rates and yields tend to be lower than predicted values.

**Table 3:** Composition, final yields (Rf) and initial conversion rates (v01) for optimized mixtures

#	Type	CBH1	CBH2	EG1	EG2	Cel74a	XYN1	**Rf pred**.	**Rf exp**.	**v01 pred**.	v01 exp
**28**	**Max Rf**	38.2	31.5	17.4	7.0	0	6.0	65.0	62.7	4.1	3.8
**29**	**Max Rf et EG1 = 7%**	40.0	39.7	7.0	7.3	0.1	6.0	62.7	62.2	3.9	3.7
**30**	**Max v01**	40.8	23.0	25.9	7.3	0	3.0	62.7	58.8	4.0	3.8

pred., predicted; exp., experimental.

**Figure 5:**
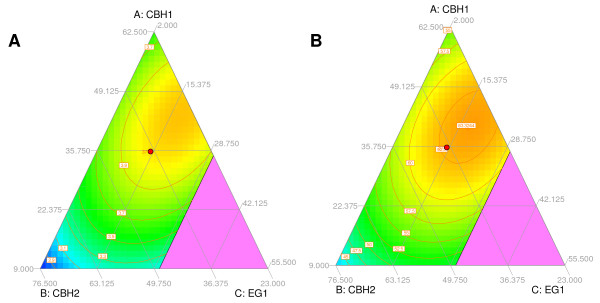
**Ternary plots showing the initial conversion rate v01 (A) and final yield (B) as a function of the three main *T. reesei *cellulases**. The other three enzymes have been fixed to the proportions of the point in the center (EG2 = 6.3%, Cel74a = 1.7% and XYN1 = 4.5%).

What is striking in the preceding results is that no points with better conversion rates or final yields than the best points of the experimental design can be found in spite of maximized responses. As an example, the center point of the model (CBH1:35.5, CBH2:37.3, EG1:14.7, EG2:6.3, Cel74a:1.7, and XYN1:4.5) has a final yield of 62.8%, very close to the experimental Rf of point 28 (62.7%) with maximized final yield. The compositions of the two points are indeed very similar suggesting that the center point lies within the optimal domain. A better representation of the optimal domain can be gained by ternary plots. In Figure 5, the proportions of minor enzymes (EG2, Cel74a and XYN1) have been set to the values of the center point and initial conversion rates and final yield are predicted as a function of varying ratios for the three major enzymes. Confirming the first analyses of model responses, the results for initial rates and final yields are similar. The position of the point in the center lies within the domain of maximal responses for yield and initial conversion rate, explaining why no other points with significant improvements can be found. As can be seen, the domain yielding optimal responses is rather large and comprises ratios for CBH1 from 35% to 50%, from 23% to 40% for CBH2 and from 13% to 25% for EG1. Concerning CBH1 and CBH2, the ratios found correspond well to those usually produced by *T. reesei *CL847 cultures on inducing carbon sources, such as lactose [4]. However, EG1 levels in these secretomes will be limiting as they are usually about 6% to 8% of total secreted proteins. Similarly, high EG1 ratios have also been found to be necessary for optimal conversion of a high variety of substrates and pretreatments by a six component mixture [19], suggesting that this enzyme is of major importance for the hydrolysis of lignocellulosic substrates in general.

These rather flexible borders for three major enzymes leading to maximized hydrolysis yields and initial conversion rates are in contrast to the narrow optimum that was obtained in the study of Gao *et al. *[20] for the lowest enzyme loading (7.5 mg/g glucan, compared to 4.3 mg/g glucan in our study). In that study, the substrate used was AFEX pretreated corn stover which might be more sensitive to changing CBH1/CBH2/EG1 ratios than steam pretreated wheat straw which is a rather easily degradable substrate [20].

### Importance of hemicellulolytic enzymes

**Figure 6:**
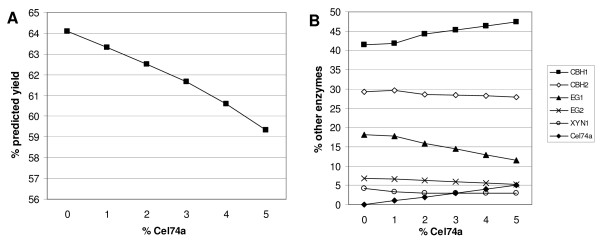
**Influence of Cel74a on predicted final yield (a) and on composition of the resulting optimized mixtures (b)**. Yields were maximized by the model with the additional constraint Cel74a = 0, 1, 2, 3, 4 or 5% of the mixture.

Model predictions for optimized final yield or initial conversion resulted in mixtures containing no or very low levels of Cel74a (see Table 3), suggesting that this enzyme is not necessary for optimal hydrolysis. Dependence of hydrolysis yield on this enzyme was further investigated and the results confirmed the negative influence of Cel74a on the final yield. As illustrated in Figure 6, highest yields are obtained when this enzyme is absent. Cel74a has xyloglucanase activity [29] and has been shown to be beneficial for the hydrolysis of steam pretreated barley straw, especially when it was pretreated under mild conditions conserving a larger percent of xylans and xyloglucans [24]. The steam-pretreated wheat straw here contains only a few percent xylan (< 3%) and it is thus probable that xyloglucanase activity is not necessary for efficient hydrolysis of this substrate. In addition, it was shown in the present and in previous studies that EG1 has high xyloglucanase side activity [24] which is probably high enough to degrade eventually xyloglucans presen tin the substrate used here. If the share of Cel74a increases in the mixture, EG1 ratios are indeed decreased by the model in order to still reach optimal yield, whereas apparently more CBH1 is necessary to maintain best activity (Figure 6b). The lower final yield obtained by including Cel74a in the enzyme mixture can thus be explained by the decrease of other more important enzymes (especially EG1 but also CBH2 and EG2) and the resulting lower overall efficiency of the cocktail.

**Figure 7:**
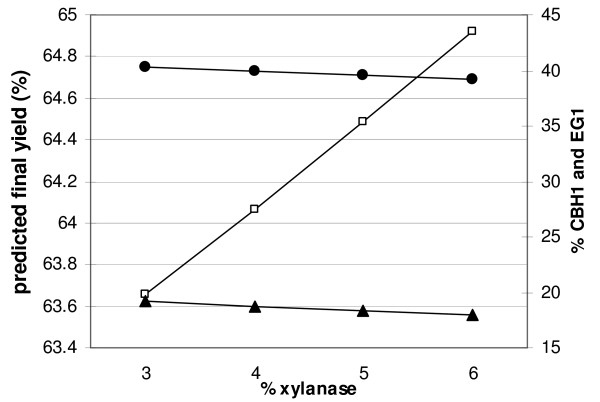
**Influence of XYN1 on the predicted final yield and on enzyme contents of optimized mixtures**. Yields (open squares) were maximized by the model with the additional constraint XYN1 = 3, 4, 5 or 6% of the mixture. Increasing xylanase levels led to a decrease of CBH1 and EG1 ratios (black circles and triangles, respectively), whereas the other three enzymes are only slightly affected (not shown).

Regarding the xylanase, the opposite case seems to apply. Conversion yields increase when the ratio of xylanase in the mixture increases and is highest at the edge of the modelled domain corresponding to a ratio of 6%, the upper limit for xylanase in the experimental design (Figure 7). This led us to hypothesize that yields could still be improved when xylanase content is increased above 6%. To test this assumption and determine the optimal ratio of xylanase, we carried out two additional hydrolysis reactions with mixtures containing 9% and 12% xylanase, respectively. These points are situated outside the modelled domain, so no reliable prediction can be made as to conversion rates or yields. Table 4 shows that point 31 with 9% xylanase indeed results in a higher hydrolysis yield than all preceding points, 66%. The hydrolysis yield of point 32 is only 61%, indicating that optimal xylanase levels must lie between 7% and 11%. The synergistic action of xylanase with cellulases has already been demonstrated in earlier studies using corn stover [15,27,30-32]. Effects were more important on AFEX-treated corn stover as this substrate has a higher xylan content, but were also clear on the dilute acid pretreated substrates having less than 6% xylan [27,31]. Mixture optimization on AFEX-treated corn stover resulted in optimal xylanase contents of 13% to 22% [20,33]. Although pretreated wheat straw has a lower xylan content than AFEX-treated material, some xylanase might also in this case be important to improve the accessibility for cellulases to their substrate. In this context, it is interesting to observe that the initial conversion rate is not higher than for other points (3.2 and 3.0). In addition, point 30, obtained by maximizing the initial conversion rate, only contains 3% xylanase, in contrast to points 28 and 29 that have maximixed final yields (Table 3). The most probable interpretation would be that in the beginning only easily accessible cellulose is degraded with no need for xylanase activity. Rather, this enzyme becomes important at later stages of hydrolysis, when xylan has to be removed to liberate obstructed cellulose.

**Table 4:** Composition and final yields (Rf) for xylanase enriched cocktails

#	Type	CBH1	CBH2	EG1	EG2	Cel74a	XYN1	**Rf pred**.	**Rf exp**.
31	xylanase +	41.4	27.4	15.9	6.3	0	9	66.2	65.8
32	xylanase ++	40	26.5	15.4	6.1	0	12	67.7	60.9

pred., predicted by extrapolation; exp, experimental.

In addition to the two hemicellulolytic enzymes, other minor enzymes present in complete *T. reesei *enzyme mixtures, which might be present in trace amounts in the purified fractions, could have synergistic action with the major enzymes and influence hydrolysis efficiency. However, considering that variations of major enzymes over a large range do not lead to important changes in optimal yield and initial conversion, the influence of trace amounts of other enzymes is likely to be negligible.

### Role of endoglucanases EG1 and EG2

**Figure 8:**
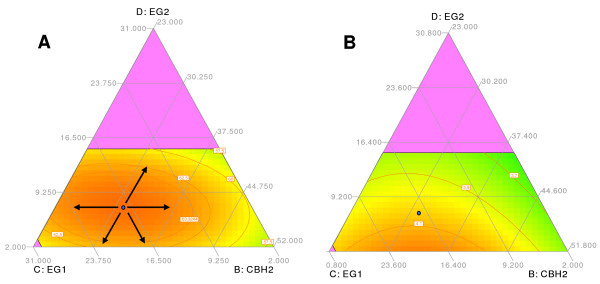
**Ternary plot showing predicted final yield (a) and initial conversion (b) as a function of CBH2, EG1 and EG2 content**. Proportions of the other enzymes were fixed according to point 28 (CBH1 = 38.2%, Cel74a = 0% and XYN1 = 6%). The blue point shows the position of point 28 and arrows indicate variations of component ratios as discussed in the text.

The ternary plot of Figure 4 shows that EG1 should account for 13% to 25% for most efficient hydrolysis, and maximization of yield resulted in point 28 with 17.4% EG1. We were interested to know if lower EG1 levels can be compensated by higher EG2 levels. The answer given by the statistical model is illustrated in the ternary plot of Figure 8. This plot shows the variation of final yield and initial conversion as a function of EG1, EG2 and CBH2 ratios when the other three compounds were set to the values of point 28. Concerning the final yield (Figure 8a), the optimal domain is a horizontal stretch parallel to the BC axis, meaning that when increasing or lowering the proportions of EG1 over a large range, a high final yield can be conserved. Similar to the findings depicted in Figure 5, the optimum yield is conserved over a range from about 13% to 23% EG1. Moving horizontally from point 28 in the optimal domain, however, does not change EG2 ratios, but only CBH2 ratios. This implies that lower EG1 levels are not compensated by EG2 which has a high specific endoglucanase activity [34,35], but rather by higher CBH2 ratios. The minor endoglucanase activity of this enzyme [23] in combination with its exoglucanase activity might contribute to the potential of CBH2 to maintain an optimal yield under the present conditions.

If EG1 levels are lowered by moving upwards from point 28 along the CD axis, then the proportion of CBH2 stays the same and EG2 increases. In this case, however, the final yield decreases much more rapidly, as the optimal domain is narrower in this dimension (optimal EG2 ratios are in the 5% to 10% range). This means that EG2 cannot compensate very well for decreasing EG1 ratios. On the other hand, when EG2 ratios are decreased from point 28 either along the CD or the DB axis, hydrolysis yields decrease slightly and in a similar way, suggesting that EG2 can (at least partially) be replaced by either EG1 or CBH2. These results point to a different function of EG1 and EG2 in the hydrolysis of steam-pretreated wheat straw.

Contrasting conclusions were drawn in the study of Banerjee *et al. *[19]. They obtained two different optimal enzyme mixtures for AFEX corn stover (one having a much higher EG1/EG2 ratio than the other) and interpreted this finding by overlapping activities of the two endoglucanases. The different structure and composition of the two substrates studied might account for the differences observed. More knowledge about the molecular structure of cellulose in different lignocellulosic substrates and the reaction mechanism of the multi-enzymatic cellulolytic complex is clearly needed to understand the role of either endoglucanase in hydrolysis of these substrates.

Interestingly, the position of the optimal domain is not the same when the initial conversion rate is considered. Figure 8b shows that the optimal domain is at the lower level of EG2 (2%), and indicates that setting EG2 to 0% could further improve the initial rate. These results suggest that EG2 can be omitted in the initial phase of hydrolysis of steam-exploded wheat straw under the present conditions, whereas a minimum of 5% would be required for best yields.

Although these findings are only the result of model predictions, it is interesting to view them in relation to results published by Szijarto *et al. *[35], who identified EG2 (Cel5a) as a key component for the liquefaction of pretreated wheat straw. This highlights the fact that different conditions (in this case it is essentially the dry matter content that varies, from 15% in the cited study to 1% in ours) may require different ratios of certain enzymes for optimal hydrolysis.

Similarly, optimal cocktail compositions may also depend on enzyme loading. The present study has been conducted with low protein loading (2.5 mg g^-1 ^dry weight (DW)) which also explains the rather low yields after 48 hours of hydrolysis (64% at the best). The study of Gao *et al. *[20] demonstrates that optimum mixtures do not change very much when the protein loading is increased fourfold. The most evident difference was that EG1 is more important at lower protein loadings, as free chain ends for cellobiohydrolase action might be limiting.

For industrial applications, higher protein loadings and substrate content than those applied in the present study are needed. It was, therefore, of interest to test an optimized mixture under such conditions. A hydrolysis experiment with the reconstituted mixture corresponding to the 'natural' K630 cocktail (Figure 1) and to the optimized point 28 was conducted at 15% DW. With 2.5 mg g^-1 ^enzyme loading, the former mixture led to a hydrolysis yield of 37 ± 1%, the latter liberated 40 ± 3% of available glucose after 48 hours. While the difference is not significant, the optimized mixture is still performing well compared to the non-optimized one. When enzyme loading was increased to 5 mg g^-1 ^at the same dry matter content, the reconstituted K630 cocktail led to a 52 ± 4% hydrolysis yield after 48 hours compared to 48 ± 3% for point 28. In this context, it is interesting to note that the main difference in the composition of these two cocktails is the EG1:EG2 ratio, which is 7:14 for the K630 mixture and 17:7 for point 28. The tendentiously higher yield obtained with the reference cocktail suggests that EG2 could become more important at higher substrate content and enzyme loadings. But more experiments are clearly required to define the best cocktail composition for high dry matter reactions and modified enzyme:substrate ratios.

It is possible that hydrolysis ratios of the optimized mixtures can still be improved when other enzyme components are added. It was shown for instance that xylanases from different families (10 and 11) act synergistically and that their simultaneous presence leads to improvement of glucose yields [18,19,36]. It can be hypothesized, however, that an additional xylanase (as well as β-xylosidase) would lead only to minor improvements on steam-exploded wheat straw, as this substrate contains only very little xylan. Another candidate for further hydrolysis improvement is Cel61a which makes up less than 1% in *T. reesei *secretomes [4] but was shown to increase yields on AFEX and AP-pretreated corn stover when it was increased to about 20% of the enzyme mixture [18,19]. In hydrolysis experiments with steam-exploded wheat straw at 1% dry matter, using a mixture of purified *T. reesei *enzymes in proportions typically found after lactose induction of *T. reesei *CL847 (CBH1 52%, CBH2 33%, EG1 and EG2 7.5% each and 250 CBU g^-1 ^β-glucosidase), supplementation with GH61a did not lead to improved hydrolysis yields. After 72 hours hydrolysis at a protein loading of 5 mg g^-1 ^dry matter, 80.7 ± 4.4% yield was obtained in the presence of 10% GH61a, compared to 83.7 ± 6.1% without GH61a. The lack of positive effect could, however, be due to the experimental conditions which might have prevented GH61a activity, as Cu^2+ ^ions and a redox-active cofactor are needed for maximal GH61a activity [37].

## Conclusions

In the present study a statistical model was set up to search for optimized enzymatic mixtures containing the *T. reesei *enzymes CBH1, CBH2, EG1, EG2, Cel74a and XYN1 for the hydrolysis of steam-exploded wheat straw. While the initial conversion rate was globally correlated to final yield, some enzymes (EG2, XYN1) were predicted to be more important in the later stages of hydrolysis under the conditions used here. The present results show that mixtures with significantly higher final yield than those representing standard *T. reesei *cocktails could not be identified with the methods applied here, but suggest that EG1 is an enzyme of major importance for optimized initial conversion rates and final yield. Future studies must show if these findings are also true for conditions of high dry matter content and protein loading, conditions which are relevant for enzymatic hydrolysis in industrial applications.

## Methods

### Substrate

Steam exploded wheat straw was used as substrate in this study. Wheat straw provided by VALAGRO (Poitiers, France) was chopped and soaked overnight in a solution of 0.04 M H_2_SO_4 _for 16 hours. After draining and pressing, the steam explosion was performed in a discontinuous autohydrolysis reactor at 20 bars and 210°C and 150 seconds residence time. The pretreated straw was washed four times, freeze-dried, and ground on a 1 mm grid with a Brabender Wiley Mill grinder. Compositional analysis conducted according to the NREL/TP-510-42618 procedure yielded a glucan content of 54.7%, 2.9% xylan, 33.2% lignin, as well as 6.1% ashes.

### Enzymes

Enzymes were produced by the strain *T. reesei *CL847, a hypercellulolytic mutant strain [38], and purified using a modified version of the purification process described by Heiss-Blanquet *et al. *[39]. After a growth phase on lactose, cellulase gene expression was induced either by lactose (for purification of CBH1, CBH2, EG1 and EG2) or by a lactose/xylose mixture inducing the secretion of xylanase and Cel74a. Enzymes were produced in a 2.5 L working volume fermenter and purified from both culture supernatants using the same two-step fast protein liquid chromatography (FPLC) method. For the preliminary purification, samples were salted out using a Hi-trap desalting column (Biorad, Marnes-la-coquette, France) and equilibrated with 25 mM imidazole-HCl buffer (pH 7.5). Chromatofocusing was performed on an ÄKTA^® ^FPLC (GE Healthcare, Chalfont St Giles, UK) using a Mono Q 5/50 GL column (GE Healthcare, Chalfont St Giles, UK) equilibrated with the initial buffer. Proteins bound (20 mg) under the initial conditions were eluted by a pH gradient (from 7.4 to 3.9) using PB74 Polybuffer (GE Healthcare, Chalfont St Giles, UK) at a constant flow rate of 0.7 ml.min^-1^. Recovered fractions were analyzed by one dimensional (ID) or 2D gel electrophoresis indicating a purity of >95%.

EG4 (GH61a) was obtained by heterologous expression in *Pichia pastoris*. The coding sequence was amplified from cDNA, fused to an α-secretion factor and a C-terminal His-tag, and inserted into the pPICZαA vector. The recombinant protein was recovered from positive clones after five days of methanol induction and purified by affinity chromatography (HisTrapTM column, GE Healthcare) connected to an Äkta FPLC (GE Healthcare, Chalfont St Giles, UK), following the manufacturer's instructions.

β-glucosidase SP188 was supplied by Novozymes (Bagsvaerd, Denmark). The cellulase enzyme cocktail GC220 was purchased from Genencor-Danisco (Rochester, NY, USA). Specific activities were determined on Avicel cellulose (Sigma-Aldrich, St Louis, MO, USA), CMC (Serva, Heidelberg, Germany), oat spelt xylan (Sigma-Aldrich) and Tamarind xyloglucan (Megazyme, Wicklow, Ireland) at 1% dry matter in 0.05 M Na-citrate buffer pH 4.9. An appropriate quantity of enzyme was added to the mixtures after 10 minutes equilibration at 50°C and the reaction stopped after 10 minutes (xyloglucan and xylan), 30 minutes (CMC) or 6 hours (Avicel) by boiling for 5 minutes. Reducing sugars were measured using the DNS (dinitrosalicylic acid) method and reading the absorbance at 540 nm [40]. Glucose was used as a standard for Avicelase and CMCase activities, whereas a xylose standard was used for xylanase and xyloglucanase assays.

### Enzymatic hydrolysis

Hydrolysis experiments were carried out in 25 ml glass bottles. A total of 100 mg of sieved and freeze-dried substrate was suspended in a total volume of 10 ml containing 50 mM citrate buffer pH 4.8 (Merck, Whitehouse Station, NJ, USA), 32 μl of tetracycline (10 g l^-1^) (Sigma-Aldrich, St Louis, MO, USA) and 24 μl of cycloheximide (10 g l^-1^) (Sigma-Aldrich, St Louis, MO, USA). The flasks were incubated at 50°C for 30 minutes before addition of β-glucosidase to a final concentration of 250 CBU g^-1 ^substrate and 2.5 mg g^-1 ^of enzyme mixture.

Flasks were incubated at 50°C and 175 rpm and samples taken at 0.5, 1, 3, 6, 24 and 48 hours. Enzymes were inactivated in boiling water for 5 minutes and supernatants analyzed for glucose, cellobiose and xylose by a HPLC ISC300 Dionex system as described by Heiss-Blanquet *et al. *[39]. One hundred percent hydrolysis corresponds to a concentration of glucose of 6.44 g l^-1^. All experiments were carried out in triplicate and were always conducted in parallel to a control experiment (in duplicate) corresponding to the central point of the experimental space (one of the seventeen hydrolysis experiments constituting the design; see Results and Discussion section). The nine repetitions of the central point also served to determine the global experimental error.

Hydrolysis experiments with the addition of GH61a were conducted in the same way, with the exception that 5 mg protein g^-1 ^dry weight were used and that reducing sugars were determined after 48 hours by the DNS method [40].

### Data analysis and modelling

The model chosen is a quadratic model described by the following equation:

$$y=\overset{6}{\underset{j=1}{\sum }}{\text{b}}_{j}x_{j}+\underset{1\leq j<k\leq 5}{\sum }{\text{b}}_{jk}x_{j}x_{k}$$

where:

- *y *is the response for which the model is computed,

- *b_j_,_jk _*are the coefficients of the model to be estimated,

- and *x_j _*are the variables of the model (designation of each *x_j _*is given in Table 2).

In the model above, the quadratic terms associated with x6 are not considered (that means that pairwise synergies between cellulases but not with xylanase are taken into account).

For estimating the coefficients, an optimal design consisting of 17 hydrolysis experiments has been computed by Design Expert^® ^(Version 8.0.6, Stat-Ease, inc., 2010). The algorithmically built design is called IV-optimal design (IV for integral variance) and seeks to minimize the integral of the prediction variance across the design space. Eleven validation points have also been generated by Design Expert^® ^to check the predictive capacity of the model. The calculation and analysis of the experimental responses for each experience was performed with the R software, after analysis of the glucose concentration at different times.

## Abbreviations

AFEX: ammonia fiber expansion; CBH: cellobiohydrolase; CMC: carboxymethylcellulose; DNS: dinitrosalicylic acid; DW: dry weight; EG: endo-β-1,4-glucanase; HPLC: high performance liquid chromatography; Rf: final yield; v01: initial conversion rate; XYN1: xylanase 1.

## Competing interests

The authors declare that they have no competing interests.

## Authors' contributions

HB carried out the hydrolysis experiments, analyses and drafted the manuscript. AF set up the experimental mixture plan, developed the statistical model and participated in manuscript writing. NLF purified and characterized enzymes, participated in study conception and corrected the manuscript. SM set up and advised on sugar analyses by HPLC. SHB conceived the study, carried out data interpretation and wrote the manuscript. All authors read and approved the final manuscript.

## Supplementary Material

Additional file 1 **ANOVA results of v01 and Rf**. Statistical ANOVA (analysis of variants) tables of coefficients for quadratic terms for the two models describing initial conversion rate v01 and final yield Rf, respectively. Click here for file
