# Supplementary material for: Optimization of a synthetic mixture composed of major Trichoderma reesei enzymes for the hydrolysis of steam-exploded wheat straw
Source: Biotechnol Biofuels. 2012 Feb 28;5:9. doi: 10.1186/1754-6834-5-9 (PMC3310832; doi:10.1186/1754-6834-5-9)
Supplement: Additional file 1 — ANOVA results of v01 and Rf. Statistical ANOVA (analysis of variants) tables of coefficients for quadratic terms for the two models describing initial conversion rate v01 and final yield Rf, respectively. [file 1754-6834-5-9-S1.DOC]

**Table 1 : ANOVA results of ν_01_. Letters represent the enzymatic components of the system (A=CBH1, B=CBH2, C=EG1, D=EG2, E=Cel74a).**

|  | **Sum of** |  | **Mean** | **F** | **p-value** |
| --- | --- | --- | --- | --- | --- |
| **Source** | **Squares** | **df** | **Square** | **Value** | **Prob > F** |
| Model | 24.15 | 15 | 1.61 | 54.62 | < 0.0001 |
| Linear Mixture | 14.30 | 5 | 2.86 | 97.05 | < 0.0001 |
| AB | 0.90 | 1 | 0.90 | 30.52 | < 0.0001 |
| AC | 0.36 | 1 | 0.36 | 12.21 | 0.0008 |
| AD | 0.05 | 1 | 0.05 | 1.82 | 0.1807 |
| AE | 0.05 | 1 | 0.05 | 1.82 | 0.1814 |
| BC | 0.17 | 1 | 0.17 | 5.78 | 0.0184 |
| BD | 0.04 | 1 | 0.04 | 1.41 | 0.2376 |
| BE | 0.05 | 1 | 0.05 | 1.67 | 0.1993 |
| CD | 0.07 | 1 | 0.07 | 2.30 | 0.1327 |
| CE | 0.04 | 1 | 0.04 | 1.40 | 0.2403 |
| DE | 0.05 | 1 | 0.05 | 1.62 | 0.2059 |
| Residual | 2.51 | 85 | 0.03 |  |  |
| Lack of Fit | 0.02 | 1 | 0.02 | 0.54 | 0.4623 |
| Pure Error | 2.49 | 84 | 0.029 |  |  |
| Cor Total | 26.6 | 100 |  |  |  |

**Table 2 : ANOVA results of Rf. Letters represent the enzymatic components of the system (A=CBH1, B=CBH2, C=EG1, D=EG2, E=Cel74a).**

|  | **Sum of** |  | **Mean** | **F** | **p-value** |
| --- | --- | --- | --- | --- | --- |
| **Source** | **Squares** | **df** | **Square** | **Value** | **Prob > F** |
| Model | 6995.37 | 15 | 466.36 | 96.34 | < 0.0001 |
| Linear Mixture | 3817.25 | 5 | 763.45 | 157.72 | < 0.0001 |
| AB | 294.37 | 1 | 294.37 | 60.81 | < 0.0001 |
| AC | 125.10 | 1 | 125.10 | 25.84 | < 0.0001 |
| AD | 40.63 | 1 | 40.63 | 8.39 | 0.0048 |
| AE | 8.24 | 1 | 8.24 | 1.70 | 0.1954 |
| BC | 82.06 | 1 | 82.06 | 16.95 | < 0.0001 |
| BD | 33.55 | 1 | 33.55 | 6.93 | 0.0101 |
| BE | 7.43 | 1 | 7.43 | 1.53 | 0.2189 |
| CD | 46.83 | 1 | 46.83 | 9.67 | 0.0025 |
| CE | 7.07 | 1 | 7.07 | 1.46 | 0.2302 |
| DE | 12.08 | 1 | 12.08 | 2.49 | 0.1180 |
| Residual | 411.46 | 85 | 4.84 |  |  |
| Lack of Fit | 1.69 | 1 | 1.69 | 0.3469302 | 0.5574 |
| Pure Error | 409.76 | 84 | 4.87814074 |  |  |
| Cor Total | 7406.83095 | 100 |  |  |  |
